# Elevated Risk of an Emergency Department Admission Associated With Long COVID Diagnosis Within the US Veteran Population

**DOI:** 10.7759/cureus.98410

**Published:** 2025-12-03

**Authors:** Raymond Van Cleve, Amanda Lienau, Jonathan Sanchez Garcia

**Affiliations:** 1 Medicine, Stanford University, Palo Alto, USA; 2 Innovation Ecosystem, Veterans Affairs, St. Louis, USA; 3 Data Analysis, MDClone Inc., Beer Sheba, ISR

**Keywords:** emergency medical service, long covid, long-term outcome, management of long covid, veterans health

## Abstract

Long COVID has emerged as a significant public health concern, potentially affecting millions of people over the next decade. By examining the risk of an emergency department (ED) visit associated with long COVID across a population, we can see the population-level severity of long COVID. This study aims to quantify the change in risk for ED visits in the six months following COVID-19 infection for people diagnosed with long COVID compared to those who are only infected with COVID-19 but not diagnosed with long COVID.

The study compared the risk of an ED visit between veterans with long COVID and those with only COVID-19 infection. We examined the risk of an ED visit for these two groups in the six months before and the six months after being infected with COVID-19. Data came from the Veterans Health Administration (VHA) electronic medical records, specifically veterans who used the Veterans Affairs (VA) healthcare system and had an initial case of COVID-19 between March 1, 2021, and December 21, 2021. We examined ER visits six months before and after testing positive for COVID-19. The outcome of interest was the risk of an ED visit for people diagnosed with long COVID compared to people who only contracted COVID-19 and were not diagnosed with long COVID.

In the six months after contracting long COVID, veterans eventually diagnosed with long COVID had a 34% higher risk of ED visits compared to those who contracted COVID-19 but never developed long COVID. Between three and six months post-infection, the risk of ED visits was 21% higher in the long COVID group. Long COVID can be a severe condition whose effects can last months after infection and diagnosis. Certain policies need to be implemented to manage the symptoms of this disease and reduce the need for emergency department services.

## Introduction

Morbidity and mortality associated with acute COVID-19 infection have declined considerably over the past three years due to increased vaccination and less dangerous variants of the virus circulating [1,2]. Unfortunately, a threat from COVID-19 infection that has persisted is post-acute sequelae of SARS-CoV-2 (PASC), also known as long-haul COVID-19 or long COVID. Long COVID is defined by the Centers for Disease Control and Prevention as persistent COVID-related symptoms three or more months after infection [3]. Previous research has found that long COVID symptoms can range in severity from mild to debilitating, and previous surveillance has identified over 200 different symptoms experienced by patients with long COVID [4-6]. Long COVID has been observed to last anywhere from several weeks to multiple months and, in some cases, multiple years [7]. While long COVID rates overall have decreased as less virulent COVID variants have begun circulating, the risk of developing long COVID from the Omicron family of COVID variants is estimated to be about 16% globally, although this estimate may differ depending on the criteria used to define and diagnose long COVID [4,8]. In the United States, most populations face a uniform risk of developing long COVID [9]. With the acute disease associated with COVID-19 entering into an endemic phase and each person facing a risk of developing long COVID, the size of the public health burden and the extent to which long COVID will affect the overall healthcare system are just beginning to be understood [10]. This paper examines the nature and size of the public health burden of long COVID, specifically within the US veteran population. This paper seeks to determine if veterans with long COVID face an increased risk of an emergency department (ED) visit compared to those who contract COVID-19 but do not develop long COVID.

A report from the Centers for Disease Control and Prevention estimates that 6.9% of all adults in the United States have ever had long COVID in some form [11]. Recent studies of the Veterans Affairs (VA) administration estimate that approximately 13.5% of the VA population has had long COVID since the onset of the pandemic [12]. Estimating the prevalence of severe or debilitating long COVID, however, is much more challenging given the lack of standardization of the disease and the myriad of ways long COVID can present itself [13]. There is still no definitive physiological explanation for long COVID, no definitive biomarker or test that can identify long COVID (aside from the presentation of symptoms) and the duration of the disease, and no formal measurement of disease severity [14,15]. While long COVID can affect every organ system, some studies have found that people with long COVID face a higher risk of pulmonary complications while they are experiencing long COVID. This research, however, has not derived the specific risk of pulmonary complications or any other complications associated with long COVID and has not been able to estimate the size of the public health burden created by long COVID [16]. Overall, there is limited and inconclusive research on how large of an impact long COVID will have on any population or the overall health consequences of long COVID that will be seen throughout society in the coming years [17,18].

Adjacent to public health, one economic estimate of one of the burdens created by long COVID examines the reduction in labor force participation as a result of long COVID. Previous research found that, as of 2022, approximately 4.5 million people have either dropped out of the workforce altogether or significantly reduced their working hours due to complications from long COVID [19]. This estimate illustrates both how problematic long COVID may be on a population level and that the problems associated with long COVID may be more complex than just extended COVID symptoms. Given this drop in the labor force as a result of long COVID, the burden created by long COVID to public health and the healthcare system may also be considerably large and unwieldy. One potential public health concern related to long COVID is an increased risk of visiting an emergency department (ED). ED visits on their own are clear markers of poor health and misery, but they may also signal greater underlying distress within a population. Individuals with long COVID frequently present to EDs due to exacerbation of symptoms such as fatigue, dyspnea, and chest pain, which are not easily managed in primary care settings [20]. Long COVID ED admissions involve problems spanning the entire body, including neurological, cardiovascular, and respiratory symptoms, which contribute to the severity of patients' conditions and their need for acute medical care [21]. These ED visits underscore the complex and often debilitating nature of long COVID and signal the larger burden of disease facing this population.

This paper seeks to better understand long COVID on a population level, specifically examining the additional risk of an emergency department visit faced by patients in the Veterans Affairs healthcare system with long COVID. The metric of emergency department visits has been used in the past to gain insight into the complexities and nature of various public health problems, including, but not limited to, mental health, violence, child abuse, drug overdoses, and acute COVID-19 infection [22-24]. This paper seeks to examine whether veterans using the VA healthcare system who have been diagnosed with long COVID face a higher risk of going to the emergency department compared to veterans who contracted COVID-19 but were not diagnosed with long COVID.

## Materials and methods

Data

The data for this study came from the Veterans Health Administration (VHA) corporate data warehouse (CDW). The VHA CDW comprised operational and health-related data for each patient seen at a VA healthcare center. We accessed the VA's CDW using MDClone, a third-party analytics tool capable of querying and analyzing VA data. The data queried for this project was initially part of an operational initiative examining long COVID trends. This project was conducted under a Cooperative Research and Development Agreement (CRADA) and followed the ethical guidelines outlined under VHA Directive 1206.

The population we examined were individuals enrolled in VA care and had tested positive for COVID-19 at a VA facility or had a case of COVID-19 confirmed by a VA clinician. The outcome variable we were interested in was an emergency department visit. Given the breadth of symptoms associated with long COVID, we analyzed all ED visits and did not limit the ED visits by cause. We examined ED visits six months prior to testing positive for COVID-19 and six months after testing positive for COVID-19. The independent variable was whether the person would eventually be diagnosed with long COVID. We classified people as being diagnosed with long COVID if they had a diagnostic code of U0.09. We also included health history information, demographic data, and data on social determinants of health as covariates. The covariates we included had initially been analyzed in prior VA long COVID analyses [12].

In order to be included in our population, the person had to test positive for COVID-19 between March 1, 2021, and December 31, 2021. We chose the starting point of this timeline because it coincided with when the VA had put together formal diagnostic criteria for post-acute sequelae of COVID-19 (also known as long COVID) and allowed time for these criteria to be disseminated throughout the VA system. The latest date for which we could reliably obtain ED visit data for the entire VA population was June 30, 2022, which determined the December 31 end date for our time frame, since we examined ED visits six months before and six months after a person tested positive for COVID-19.

To ensure precision within our population, we only included people for whom the COVID-19 case we were examining was their first case of COVID-19. If a person had a COVID-19 infection prior to our time frame, that person was not included in the study population.

Analysis

The first analysis conducted was descriptive and examined the demographic, social, and healthcare-related qualities between those who were eventually diagnosed with long COVID and those who were infected with COVID-19 but were never diagnosed with long COVID. The computational analysis was done using MDClone ADAMS (2024) [25].

The second analysis sought to examine whether a person diagnosed with long COVID faced a higher risk of going to the emergency department after being infected with COVID-19. When comparing the risk of an ED visit among people diagnosed with long COVID and those not diagnosed, there is an inherent possible bias. People diagnosed with long COVID may have poorer overall health and/or a weaker immune system. Given this, these individuals may already have an elevated risk of an ED visit, separate from having long COVID. While this bias cannot completely be overcome, some approaches can partially isolate the elevated risk of an ED visit associated with being diagnosed with long COVID.

**Figure 1:**
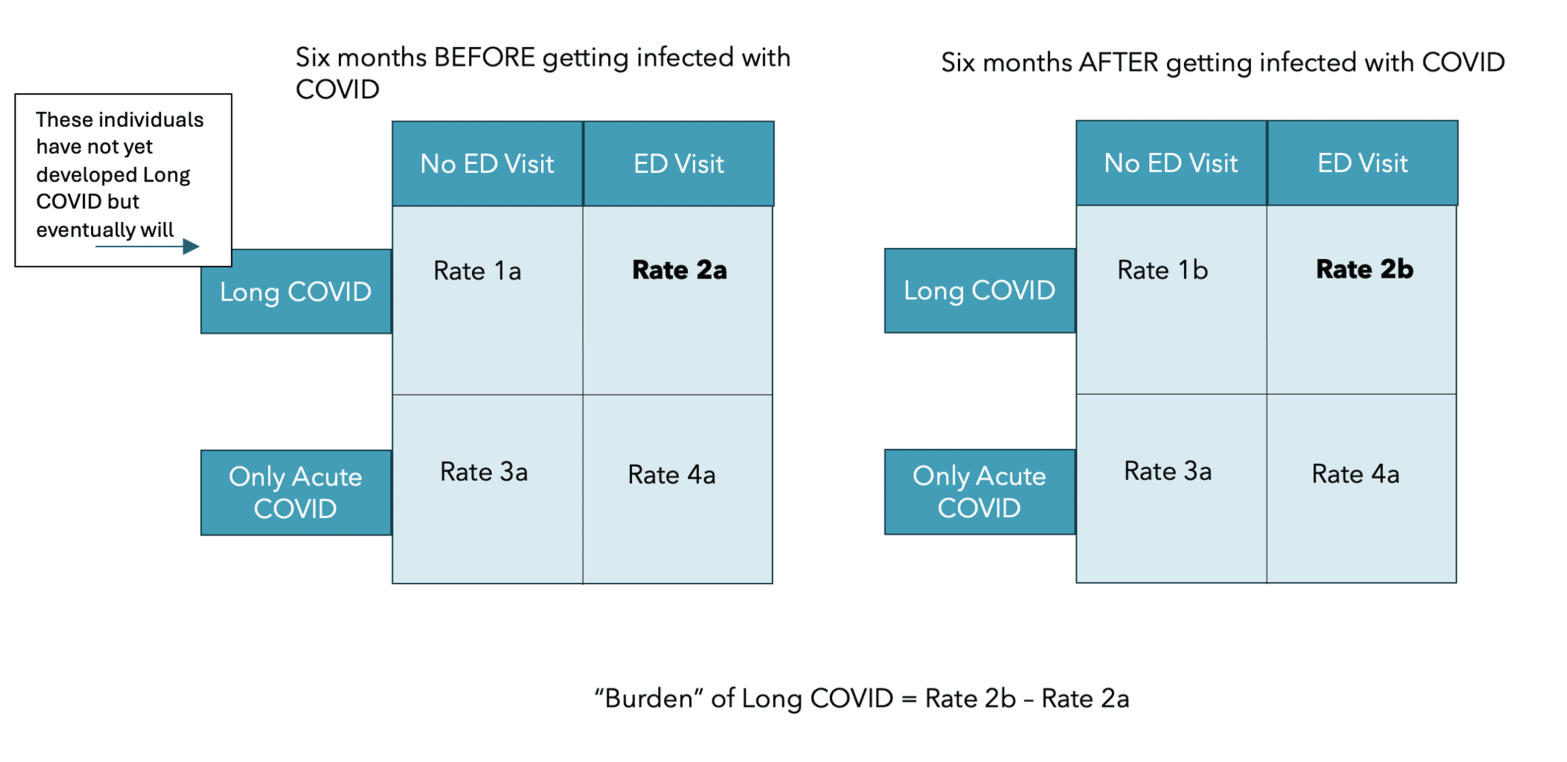
Conceptual design of our study ED: emergency department

To manage this bias, we compared a person's risk of an ED visit before and after contracting COVID-19 and then examined the difference in the risk of an ED visit between those who eventually would be diagnosed with long COVID and those who would only be infected with COVID-19 and would not be diagnosed with long COVID. In effect, we are asking what is the increase in risk for an ED visit that a person faces before and after becoming infected with COVID-19, and is that increase in risk the same if a person eventually develops long COVID? We examined the risk of an ED visit within the six months prior to becoming infected with COVID-19 and sought to observe the difference in risk between those only infected with COVID-19 and those who were infected with COVID-19 and subsequently diagnosed with long COVID. We then conducted the same analysis, except we examined the risk of an ED visit for those two groups in the six months after being infected with COVID-19. Figure 1 graphically explains this analytical plan using epidemiological 2x2 tables. This analysis was inspired by difference-in-difference models, although the model that was eventually employed was not a difference-in-difference model.

We also examined the increased risk in an ED visit for people with and without a long COVID diagnosis between three and six months after they tested positive for COVID-19, attempting to see if the elevated risk in ED visits is experienced beyond the initial infection period. Long COVID is generally classified as experiencing COVID-19-related symptoms at least three months after being infected with COVID-19 [26]. By examining the difference in risk for an ED visit between three and six months after testing positive for COVID-19, we can understand if an elevated risk of an ED visit is experienced in the long COVID period or if it is experienced closer to the time of infection.

The elevated risk of an ED visit associated with long COVID was analyzed using logistic regression. The outcome variable was whether or not a person experienced an ED visit during the specified time period (either 0-6 months or 3-6 months after infection). The independent variable was whether or not a person was (or would eventually be) diagnosed with long COVID. Other demographic, social, and health-related variables were included as covariates. This analysis included three logistic regression models; the first model examined the risk of experiencing an ED visit in the six months before a person was infected with COVID, the second model examined the risk of experiencing an ED visit in the six months after a person was infected with COVID, and the third model examined the risk of experiencing an ED visit 3-6 months after a person was infected with COVID.

## Results

**Figure 2:**
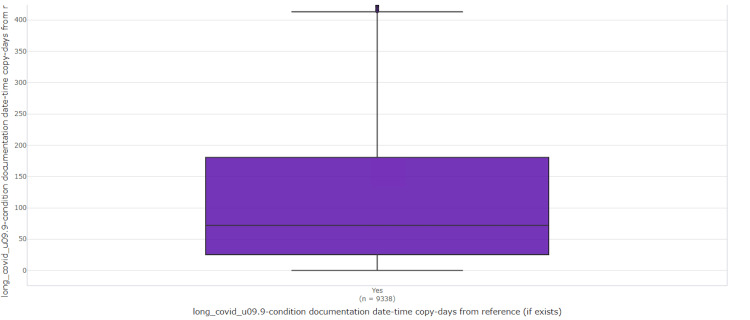
Average number of days from the first positive COVID test to a diagnosis of long COVID

Our population consisted of 172,945 VHA patients who tested positive for COVID-19 or had a confirmed case of COVID-19, 9,338 of whom eventually were diagnosed with long COVID (Table 1). The average age of our population was between 58 and 59 years old, while the average age of people who were diagnosed with long COVID was between 61 and 62 years old. Our population was composed of 85% male and 15% female patients; 67% were white, 19% black, 13% identified as other when asked about their racial category, and 1% Asian. In terms of urban or rural status, 69% lived in urban areas, 29% in rural areas, and 1% in very rural areas (Table 1). People diagnosed with long COVID had a chi-squared value of less than 0.001 for every demographic, social, and health-related category (Table 1). Of those diagnosed with long COVID, the average (mean) number of days a long COVID diagnosis was made after initially testing positive for COVID-19 was 108 days (standard deviation (SD): 98.7 days) (Figure 2).

**Table 1:** Demographic and health-related information SD: standard deviation, COPD: chronic obstructive pulmonary disease, PTSD: post-traumatic stress disorder

Characteristics	Overall		Only infected with COVID-19		Infected with COVID-19 and developed long COVID		Chi-squared	P-value
	Number	SD or %	Number	SD or %	Number	SD or %		
Total population	172,945	-	163,607	-	9,338	-	-	-
Diagnosed with long COVID	9,338	5%	0	0	9,338	100%	-	-
Age	58.81	16.54	58.67	16.63	61.32	14.73	<0.001	<0.001
Gender							<0.001	<0.001
Male	146,284	85%	138,233	84%	8,051	86%	-	-
Female	26,661	15%	25,374	16%	1,287	14%	-	-
Race							<0.001	<0.001
Asian	1,670	1%	1,565	1%	105	1%	-	-
Black	32,565	19%	31,044	19%	1,521	16%	-	-
Other	23,103	13%	22,180	14%	923	10%	-	-
White	115,607	67%	108,818	67%	6,789	73%	-	-
Ethnicity							<0.001	0.03
Declined to answer	2,205	1%	2,081	1%	124	1%	-	-
Hispanic or Latino	13,978	8%	12,808	8%	1,170	13%	-	-
Not Hispanic or Latino	134,459	78%	127,146	78%	7,313	78%	-	-
Unknown to the patient	2,502	1%	2,369	1%	133	1%	-	-
Marital status							<0.001	<0.001
Divorced	37,561	22%	35,402	22%	2,159	23%	-	-
Married	87,095	50%	81,811	50%	5,284	57%	-	-
Never married	26,480	15%	25,343	15%	1,137	12%	-	-
Separated	6,157	4%	5,858	4%	299	3%	-	-
Single	350	0%	339	0%	11	0%	-	-
Unknown	6,928	4%	6,840	4%	88	1%	-	-
Widowed	6,559	4%	6,218	4%	341	4%	-	-
Urban or rural status							<0.001	<0.001
Highly rural	1,563	1%	1,457	1%	106	1%	-	-
Rural	49,842	29%	46,878	29%	2,964	32%	-	-
Urban	120,155	69%	113,954	70%	6,201	66%	-	-
Health-related conditions								
Diabetes at the time of infection	73,452	42%	68,738	42%	4,714	50%	<0.001	<0.001
Myocardial infarction at the time of infection	10,733	6%	9,975	6%	758	8%	<0.001	<0.001
COPD at the time of infection	39,158	23%	36,231	22%	2,927	31%	<0.001	<0.001
PTSD at the time of infection	57,418	33%	53,859	33%	3,559	38%	<0.001	<0.001
Opioid prescription at the time of infection	51,567	30%	48,135	29%	3,432	37%	<0.001	<0.001
Congestive heart failure at the time of infection	22,373	13%	20,727	13%	1,646	18%	<0.001	<0.001
Chronic kidney disease at the time of infection	28,745	17%	26,835	16%	1,910	20%	<0.001	0.01
Cardiovascular disease at the time of infection	19,873	11%	18,546	11%	1,327	14%	<0.001	<0.001
Peripheral artery disease at the time of infection	13,311	8%	12,378	8%	933	10%	<0.001	<0.001
Ventricular tachycardia at the time of infection	9,199	5%	8,317	5%	882	9%	<0.001	<0.001
Obstructive sleep apnea at the time of infection	62,079	36%	57,670	35%	4,409	47%	<0.001	<0.001
Depression at the time of infection	58,967	34%	55,047	34%	3,920	42%	<0.001	<0.001
Bipolar at the time of infection	8,731	5%	8,284	5%	447	5%	0.245	0.4
Schizophrenia at the time of infection	2,874	2%	2,748	2%	126	1%	0.017	0.02
Obesity hypoventilation syndrome at the time of infection	1,665	1%	1,518	1%	147	2%	<0.001	<0.001
Healthcare-related Interactions								
Average number of emergency department encounters before COVID infection	0.41	1.08	0.41	1.08	0.48	1.12	<0.001	<0.001
Average number of emergency department encounters after COVID infection	0.37	1.13	0.36	1.13	0.52	1.2	<0.001	0.01
Average number of any patient encounters pre-COVID	37.48	82.28	37.14	77.14	43.47	75.85	<0.001	<0.001
Average number of any patient encounters post-COVID	36.1	79.91	34.95	77.14	63.48	114.86	<0.001	<0.001

When examining people's risk of an emergency department visit in the six months prior to being infected with COVID-19, people who were infected with COVID-19 and would eventually develop long COVID were not more or less likely (p=0.22) to experience an emergency department visit compared to those who were only infected with COVID-19 and would not develop long COVID (Table 2). In the six months after a person was infected with COVID-19, people who developed long COVID were 34% more likely (p<0.001) to experience an ED visit compared with those infected with COVID-19 but who never developed long COVID. When examining the risk of an ED visit 3-6 months after a person is infected with COVID-19, people with long COVID are still 21% (p<0.001) more likely to have an ED visit compared to those who were only infected with COVID-19 (Table 3).

**Table 2:** Regressions examining risk of an ED visit 1-6 months before and 1-6 months after being infected with COVID-19 ED: emergency department, CI: confidence interval, COPD: chronic obstructive pulmonary disease

Characteristics	1-6 months before being infected with COVID-19						1-6 months after being infected with COVID-19					
	Odds ratio	Standard error	Lower 95% CI	Upper 95% CI	P-value	Chi-squared	Odds ratio	Standard error	Lower 95% CI	Upper 95% CI	P-value	Chi-squared
Long COVID status (did the person eventually develop long COVID)	-	-	-	-	-	-	-	-	-	-	-	-
Eventually develop long COVID	1.03	0.03	0.98	1.09	0.22	-	1.34	0.04	1.27	1.41	<0.001	-
Conditions at the time of infection or within a year of infection	-	-	-	-	-	-	-	-	-	-	-	-
Opioid prescription	1.74	0.01	1.69	1.79	<0.001	10 200.73	1.67	0.02	1.62	1.72	<0.001	8118.68
Diabetes	0.94	0.01	0.92	0.97	<0.001	1 296.68	0.94	0.01	0.91	0.96	<0.001	939.68
Congestive heart failure	1.26	0.02	1.22	1.31	<0.001	4 390.24	1.24	0.02	1.19	1.29	<0.001	3306.81
Chronic kidney disease	1.13	0.02	1.10	1.17	<0.001	2 697.93	1.07	0.02	1.03	1.11	<0.001	1804.14
Other cardiovascular disease	1.08	0.02	1.04	1.12	<0.001	2 008.92	1.05	0.02	1.01	1.09	0.01	1461.13
Peripheral arterial disease	1.08	0.02	1.04	1.13	<0.001	1 800.70	1.04	0.02	0.99	1.09	0.09	1232.45
Ventricular tachycardia	1.29	0.02	1.23	1.36	<0.001	1 460.09	1.21	0.03	1.15	1.27	<0.001	1067.54
Obstructive sleep apnea	1.12	0.01	1.10	1.15	<0.001	1 268.70	1.13	0.01	1.11	1.17	<0.001	1118.64
Depression	1.36	0.01	1.33	1.40	<0.001	3 723.28	1.37	0.01	1.34	1.41	<0.001	3463.34
Bipolar	1.36	0.03	1.29	1.43	<0.001	1 084.30	1.47	0.03	1.40	1.54	<0.001	1222.01
Schizophrenia	1.17	0.04	1.08	1.27	<0.001	633.93	1.18	0.04	1.09	1.28	<0.001	616.21
Obesity hypoventilation syndrome	1.23	0.05	1.11	1.37	<0.001	437.35	1.15	0.06	1.03	1.28	0.01	320.83
Myocardial infarction	1.31	0.02	1.25	1.37	<0.001	2 865.32	1.26	0.02	1.20	1.32	<0.001	2125.64
COPD	1.13	0.02	1.09	1.16	<0.001	3 137.38	1.10	0.02	1.07	1.14	<0.001	2339.87
Antidepressant prescription	1.50	0.02	1.45	1.55	<0.001	10 030.68	1.44	0.02	1.40	1.49	<0.001	8300.16
Statins	1.54	0.02	1.49	1.59	<0.001	9 781.28	1.49	0.02	1.44	1.54	<0.001	7585.54
Race	-	-	-	-	-	2 447.05	-	-	-	-	-	2228.13
Black	1.43	0.07	1.26	1.64	<0.001	-	1.57	0.07	1.36	1.81	<0.001	-
Other	0.98	0.07	0.86	1.13	0.80	-	1.05	0.07	0.91	1.21	0.52	-
White	1.00	0.07	0.88	1.15	0.95	-	1.12	0.07	0.97	1.29	0.12	-
Urban or rural	-	-	-	-	-	942.86	-	-	-	-	-	966.43
Rural	1.46	0.08	1.25	1.71	<0.001	-	1.48	0.08	1.26	1.74	<0.001	-
Urban	2.08	0.08	1.78	2.42	<0.001	-	2.12	0.08	1.81	2.50	<0.001	-
Marital status	-	-	-	-	-	2 427.20	-	-	-	-	-	2074.94
Married	0.81	0.02	0.78	0.83	<0.001	-	0.81	0.02	0.79	0.84	<0.001	-
Never married	1.12	0.02	1.07	1.16	<0.001	-	1.14	0.02	1.09	1.18	<0.001	-
Separated	1.10	0.03	1.03	1.17	0.005	-	1.12	0.03	1.05	1.19	<0.001	-
Single	0.78	0.15	0.58	1.04	0.09	-	0.97	0.14	0.73	1.28	0.81	-
Unknown	0.33	0.06	0.30	0.37	<0.001	-	0.39	0.06	0.35	0.44	<0.001	-
Widowed	0.94	0.03	0.89	1.01	0.07	-	0.92	0.03	0.86	0.98	0.01	-
Female gender (male for reference category)	1.02	0.02	0.98	1.06	0.32	329.02	1.00	0.02	0.96	1.04	0.98	202.64
Intercept	0.07		0.06	0.09	<0.001	-	0.06	0.11	0.05	0.07	<0.001	-

**Table 3:** Regressions examining the risk of an ED visit 1-6 months before and 3-6 months after being infected with COVID-19 ED: emergency department, CI: confidence interval, COPD: chronic obstructive pulmonary disease

Characteristics	1-6 months before being infected with COVID-19						3-6 months after being infected with COVID-19					
	Odds ratio	Standard error	Lower 95% CI	Upper 95% CI	P-value	Chi-squared	Odds ratio	Standard error	Lower 95% CI	Upper 95% CI	P-value	Chi-squared
Long COVID status (did the person eventually develop long COVID)	-	-	-	-	-	-	-	-	-	-	-	-
Eventually develop long COVID	1.03	0.03	0.98	1.09	0.22	-	1.21	0.04	1.14	1.28	<0.001	-
Conditions at the time of infection or within a year of infection	-	-	-	-	-	-	-	-	-	-	-	-
Opioid prescription	1.74	0.01	1.69	1.79	<0.001	10 200.73	1.62	0.02	1.57	1.68	<0.001	5582.98
Diabetes	0.94	0.00	0.92	0.97	<0.001	1 296.68	0.94	0.02	0.91	0.97	<0.001	638.19
Congestive heart failure	1.26	0.00	1.22	1.31	<0.001	4 390.24	1.21	0.02	1.16	1.27	<0.001	2292.86
Chronic kidney disease	1.13	0.00	1.10	1.17	<0.001	2 697.93	1.05	0.02	1.01	1.09	0.01	1210.28
Other cardiovascular disease	1.08	0.00	1.04	1.12	<0.001	2 008.92	1.06	0.02	1.02	1.11	0.004	1042.56
Peripheral arterial disease	1.08	0.00	1.04	1.13	<0.001	1 800.70	1.05	0.03	1.00	1.11	0.04	855.73
Ventricular tachycardia	1.29	0.01	1.23	1.36	<0.001	1 460.09	1.19	0.03	1.13	1.25	<0.001	761.75
Obstructive sleep apnea	1.12	0.00	1.10	1.15	<0.001	1 268.70	1.15	0.02	1.12	1.19	<0.001	825.09
Depression	1.36	0.01	1.33	1.40	<0.001	3 723.28	1.35	0.02	1.30	1.39	<0.001	2487.74
Bipolar	1.36	0.01	1.29	1.43	<0.001	1 084.30	1.46	0.03	1.39	1.55	<0.001	996.48
Schizophrenia	1.17	0.01	1.08	1.27	<0.001	633.93	1.19	0.05	1.09	1.30	<0.001	498.39
Obesity hypoventilation syndrome	1.23	0.01	1.11	1.37	<0.001	437.35	1.11	0.06	0.98	1.24	0.09	214.66
Myocardial infarction	1.31	0.01	1.25	1.37	<0.001	2 865.32	1.25	0.03	1.19	1.31	<0.001	1520.2
COPD	1.13	0.00	1.09	1.16	<0.001	3 137.38	1.10	0.02	1.07	1.14	<0.001	1630.48
Antidepressant prescription	1.50	0.00	1.45	1.55	<0.001	10 030.68	1.41	0.02	1.36	1.46	<0.001	5870.99
Statins	1.54	0.00	1.49	1.59	<0.001	9 781.28	1.41	0.02	1.35	1.46	<0.001	5034.63
Race	-	0.00	-	-	-	2 447.05	-	-	-	-	-	1650.81
Black	1.43	0.01	1.26	1.64	<0.001	-	1.49	0.08	1.27	1.76	<0.001	-
Other	0.98	0.00	0.86	1.13	0.80	-	1.00	0.09	0.84	1.18	0.99	-
White	1.00	0.00	0.88	1.15	0.95	-	1.06	0.08	0.90	1.24	0.50	-
Urban or rural	-	0.00	-	-	-	942.86	-	-	-	-	-	699.34
Rural	1.46	0.01	1.25	1.71	<0.001	-	1.55	0.10	1.27	1.89	<0.001	-
Urban	2.08	0.02	1.78	2.42	<0.001	-	2.19	0.10	1.80	2.67	<0.001	-
Marital status	-	0.00	-	-	-	2 427.20	-	-	-	-	-	1498.01
Married	0.81	0.00	0.78	0.83	<0.001	-	0.81	0.02	0.78	0.84	<0.001	-
Never married	1.12	0.00	1.07	1.16	<0.001	-	1.12	0.02	1.07	1.17	<0.001	-
Separated	1.10	0.00	1.03	1.17	0.005	-	1.10	0.04	1.02	1.18	0.01	-
Single	0.78	0.01	0.58	1.04	0.09	-	0.86	0.17	0.61	1.20	0.37	-
Unknown	0.33	0.02	0.30	0.37	<0.001	-	0.35	0.07	0.31	0.41	<0.001	-
Widowed	0.94	0.00	0.89	1.01	0.07	-	0.91	0.04	0.84	0.98	0.01	-
Female gender (male for reference category)	1.02	0.00	0.98	1.06	0.32	329.02	0.98	0.02	0.94	1.02	0.33	109.22
Intercept	0.07	-	0.06	0.09	<0.001	-	0.04	-	0.03	0.05	<0.001	-

## Discussion

Our paper finds that people with long COVID are notably more likely to have an ED visit in the six months after being infected with COVID-19 compared with those who were infected with COVID-19 but were never diagnosed with long COVID. This increased risk for an emergency department visit also persists at least three months after initially being infected with COVID-19. This increase in risk for an ED visit is concerning in and of itself (going to the emergency department results from a decent amount of pain and distress), but it is also a signal of the overall poorer health and greater level of discomfort and misery experienced by patients with long COVID. For every person who goes to the emergency department, there are many more at home experiencing symptoms that are quite limiting but do not merit hospitalization. Our findings show that the symptoms from long COVID are notably persistent and burdensome, and underscore the previous findings on the severity of long COVID.

One important insight into the nature of long COVID presented by this research is the duration of the disease. We see that, even three months after being infected with COVID-19 and generally after having been diagnosed with long COVID, people with this condition still face a 21% increased risk of an ED visit. These ED visits could be a result of continued stress on specific organs or organ systems; this could also be the result of a change in symptoms severity. People with long COVID can face non-linear changes in symptom severity over time [26], with symptoms getting worse after improving. These findings suggest that, for a fraction of the long COVID population, this may be the case. Future research tracking the nature and severity of symptoms is needed to further understand this progression and the specific kind of damage long COVID can do.

There is no definitive explanation of long COVID, nor is there a specific treatment that has been found to support all cases of long COVID [27]. Some previous research, however, has found that targeted rehabilitation for the affected organ systems can offer some support, as well as repurposed medication and other treatments [16,28]. This paper's findings on the longevity of severe symptoms suggest that continued monitoring of symptoms and support for the various organ systems affected may be an important treatment regardless of the physiological causes underlying long COVID.

These findings identify a need for future long COVID research. Given the increased risk for an ED visit, understanding how long COVID progresses, the different types of symptoms, and the changes in severity is paramount to alleviating the public health burden associated with long COVID. Future research also needs to examine possible interventions, specifically what kinds of primary care and outpatient care can support people with long COVID so they can avoid going to the hospital and ideally alleviate the distress from their symptoms. This paper also identifies the need to understand possible long-term damage to different organs and organ systems in the body that can be caused by long COVID.

Limitations

This paper has some limitations. The most notable limitation is that this is an observational study. While we improved the precision of this study by comparing the risk of an ED visit between people with long COVID and those who were only infected with COVID-19, there still could be variables that were not identified, and we can only say that we see an increased associated risk of an ED visit; this is not definitely causal. Another limitation is that some studies of long COVID suggest that it may cause an onset of other conditions [29]. This study did not look at the cause behind the ED visit, and therefore, we cannot say how much of a factor long COVID was overall compared to other conditions. The final limitation is that we examined people enrolled in VA healthcare, which is different from the general public.

## Conclusions

While long COVID has received less attention recently, it poses a considerable threat to public health for the US veteran and the general US population. These findings identify a signal that people with long COVID face a much higher risk of going to the emergency department, and that risk continues even months after being infected with COVID-19. This increased risk of ED visits signals that people with long COVID are continuing to struggle with the weighty burden of long COVID symptoms and are at risk for potential long-term health problems. This paper demonstrates the need for continued medical care for people with long COVID. As future long COVID policy is developed, ideally, recommendations will be made so that people can utilize primary and outpatient care to manage their symptoms and support their health, and do not need to turn to the emergency department.
